# MicroRNAs as Potential Pharmaco-Targets in Ischemia-Reperfusion Injury Compounded by Diabetes

**DOI:** 10.3390/cells8020152

**Published:** 2019-02-12

**Authors:** Hassan Dehaini, Hussein Awada, Ahmed El-Yazbi, Fouad A. Zouein, Khodr Issa, Assaad A. Eid, Maryam Ibrahim, Adnan Badran, Elias Baydoun, Gianfranco Pintus, Ali H. Eid

**Affiliations:** 1 Department of Pharmacology and Toxicology, Faculty of Medicine, American University of Beirut, Beirut P.O. Box 11-0236, Lebanon; had29@mail.aub.edu (H.D.); ae88@aub.edu.lb (A.E.-Y.); fz15@aub.edu.lb (F.A.Z.); ki12@aub.edu.lb (K.I.); mki04@mail.aub.edu (M.I.); 2 Department of Biology, American University of Beirut, Beirut P.O. Box 11-0236, Lebanon; hma86@mail.aub.edu (H.A.); eliasbay@aub.edu.lb (E.B.); 3 Department of Pharmacology and Toxicology, Alexandria University, Alexandria P.O. Box 21521, El-Mesallah, Egypt; 4 Department of Anatomy, Cell Biology and Physiological Sciences, Faculty of Medicine, American University of Beirut, Beirut P.O. Box 11-0236, Lebanon; ae49@aub.edu.lb; 5 Department of Nutrition, University of Petra, Amman P.O Box 961343 Amman, Jordan; abadran@uop.edu.jo; 6 Department of Biomedical Sciences, College of Health Sciences, Qatar University, Doha P.O. Box 2713, Qatar; 7 Biomedical Research Center, Qatar University, Doha P.O. Box 2713, Qatar

**Keywords:** pharmaco-targets, diabetes, ischemia-reperfusion injury, microRNA, reactive oxygen species, apoptosis

## Abstract

Background: Ischemia-Reperfusion (I/R) injury is the tissue damage that results from re-oxygenation of ischemic tissues. There are many players that contribute to I/R injury. One of these factors is the family of microRNAs (miRNAs), which are currently being heavily studied. This review aims to critically summarize the latest papers that attributed roles of certain miRNAs in I/R injury, particularly in diabetic conditions and dissect their potential as novel pharmacologic targets in the treatment and management of diabetes. Methods: PubMed was searched for publications containing microRNA and I/R, in the absence or presence of diabetes. All papers that provided sufficient evidence linking miRNA with I/R, especially in the context of diabetes, were selected. Several miRNAs are found to be either pro-apoptotic, as in the case of miR-34a, miR-144, miR-155, and miR-200, or anti-apoptotic, as in the case of miR-210, miR-21, and miR-146a. Here, we further dissect the evidence that shows diverse cell-context dependent effects of these miRNAs, particularly in cardiomyocytes, endothelial, or leukocytes. We also provide insight into cases where the possibility of having two miRNAs working together to intensify a given response is noted. Conclusions: This review arrives at the conclusion that the utilization of miRNAs as translational agents or pharmaco-targets in treating I/R injury in diabetic patients is promising and becoming increasingly clearer.

## 1. Introduction

Ischemia-Reperfusion (I/R) injury remains a major contributor to two leading causes of death worldwide, ischemic heart disease and stroke [1]. I/R injury results from re-oxygenating tissues that had been deprived of sufficient oxygen (O_2_) [2]. The hypoxic state that arises due to ischemic conditions renders tissues more sensitive to O_2_ once circulation is restored. O_2_ stimulates endothelial cells (ECs), which consequentially produce less nitric oxide (NO) than is needed for cardiovascular homeostasis, thus increasing levels of reactive oxygen species (ROS) [3,4]. ROS are O_2_-containing free radicals which can precipitate necrosis or apoptosis by damaging cellular DNA, proteins, and lipids [5]. Moreover, the O_2_-stimulated ECs lose some of their structural integrity, allowing leukocytes to extravasate and start a tissue damage-enticed inflammatory response, accompanied by increased ROS, thus aggravating the I/R injury [6]. 

Significantly, the risk for stroke and myocardial infarction (MI) and the ensuing I/R injury is particularly high in diabetic patients [7,8]. Diabetes mellitus (DM) is the seventh leading cause of global death [9] with more than 400 million cases of diabetes estimated in 2017 [10]. DM is a chronic disease that is either due to decreased production of insulin, or increased insulin resistance; the former is referred to as type 1 DM (T1DM), and the latter is categorized as type 2 DM (T2DM). Diabetic patients suffer from prolonged hyperglycemia and periods of glucose fluctuations, which have adverse effects on the body, such as the worsening of an I/R injury. The mechanisms through which DM influences I/R injury are largely variable. However, the role of microRNAs (miRNAs) in mediating the link between DM and I/R injury, particularly as pertains to the cardiovasculature, is receiving increased attention but remains far from being fully understood. This review responds to a much-desired need for reviewing and discussing the current literature on such a role.

## 2. MicroRNAs (miRNAs)

MicroRNAs are noncoding, single-stranded RNA, ranging from 20 to 22 nucleotides in length. Their biogenesis starts when primary miRNAs (pri-mRNAs) are synthesized in the nucleus [11]. These double-stranded pri-mRNAs are cleaved to shorter hairpin RNAs called pre-miRNAs [12]. Pre-miRNAs are further cleaved to shorter double-stranded miRNAs, which then associate with the Argonaute family of proteins to form RNA-induced silencing complexes or RISC. It is in these complexes that mature miRNAs, now single-stranded, is retained [13,14]. MiRNAs play important roles in regulating gene expression by acting as a silencer of messenger RNA (mRNA). By binding to it, miRNA causes the destabilization or cleavage of pseudo-complementary sequences of target mRNA, thus inhibiting its translation [15]. This mechanism was shown to be implicated in many cellular processes such as cell metabolism, division, differentiation, apoptosis, and autophagy [16].

The interest in miRNA as a regulator of many diseases has gained momentum, especially in the cardiovascular field. Extensive research is being conducted to investigate the association of miRNA with CVD and DM. Indeed, the involvement of miRNA, both in detrimental cascades and as potential therapy options affecting angiogenesis, atherosclerosis, hypertension, and ischemic hypoxia, has advanced to the frontline of cardiovascular research [17,18]. Several mechanisms are implicated in the alteration of miRNA expression in diabetes leading to vascular complications including epigenetic DNA-methylation mechanisms affecting miRNA transcription, splicing of intronic miRNA, and disruption of transcription factor sites [19]. In this review, we aim to highlight the role of multiple miRNAs in ROS regulatory pathways in I/R injury, taking the impact of DM into account (Table 1).

### 2.1. MiR-34a

**Figure 1:**
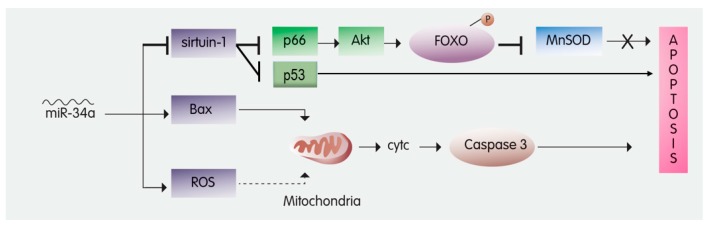
The role of miR-34a in inducing apoptosis—miR-34a induces apoptosis by inhibiting Sirtuin-1. Sirtuin-1 attenuates apoptosis by: 1—Inhibiting p66, an activator of Akt; Akt phosphorylates FOXO to inhibit MnSOD, which usually inhibits apoptosis. 2—inhibiting the transcription of p53, which usually induces apoptosis. In addition, miR-34a leads to Bax upregulation and reactive oxygen species (ROS) accumulation, hence triggering the release of Cytochrome C from the mitochondria, which activates Caspase 3 to induce apoptosis. FOXO3: Forkhead box O3; MnSOD: Manganese Superoxide Dismutase; Akt: protein kinase B; cytc: Cytochrome c.

Recent studies showed that 316 miRNAs had their expression dysregulated by diabetes in a streptozotocin-induced T1DM mouse model. Among these dysregulated miRNAs is miR34a, which is known to play a role in apoptosis, autophagy, and oxidative stress [20,21,22] (Table 1). MiR-34a is reported to silence the mRNA of silent mating type information regulation 2 homolog-1 (sirtuin-1) [23]. Sirtuin-1 is a deacetylase that acts on numerous proteins, thus regulating several cellular functions, including apoptosis (Figure 1) [24]. Interestingly, sirtuin-1 protects cardiomyocytes from I/R-induced apoptosis [25,26,27], whereas cardiomyocyte-specific deletion of Sirt1 renders the myocardium critically sensitive to I/R injury [28]. 

There are at least two modes of action through which sirtuin-1 downregulates I/R-induced apoptosis. The first pathway is by inhibiting adaptor protein p66 [29], hence activating forkhead box O3a (FOXO3a). FOXO3a is a transcription factor of manganese superoxide dismutase (MnSOD), a ROS scavenging enzyme (Figure 1) [30]. Alternatively, sirtuin-1 may inhibit the transcriptional activity of p53, thus attenuating apoptosis (Figure 1) [31,32]. 

Due to its inhibitory effect on sirtuin-1, miR-34a exacerbates myocardial injury by increasing infarct size and promoting apoptosis [26] (Table 1). Importantly, downregulating miR-34a was shown to attenuate I/R injury by inhibiting apoptosis [33]. Indeed, delivering a miR-34a mimic to neonatal hearts significantly inhibited proliferation of cardiomyocytes following MI [34]. Furthermore, blocking miR-34a improved post-MI remodeling, likely via modulating levels of Sirt1, Cyclin D1, and Bcl2 [34]. In DM, miR-34a levels increase by up to five-fold, yet insulin treatment fails to attenuate this dramatic increase miR-34a. [29,35]. Thus, it would not be surprising if DM exacerbates I/R injury due to high miR-34a levels. As such, one can envision that approaches which seek to reduce miR-34a could be used to attenuate I/R injury, as recently suggested [36].

The role of miR-34a in senescence and autophagy is becoming increasingly noted. In senescent endothelial cells, as well as in organs of aged mice, miR-34a appears to be upregulated [37,38,39] (Table 1). Equally importantly, overexpressing miR-34a is sufficient to induce senescence of cells that play an important role in I/R injury, such as endothelial cells or pro-angiogenic cultured progenitor cells [40]. It appears that the autophagy target of miR34 is Atg9 [41]. Very recently, direct evidence for Atg9 in mitochondrial health and cardiac function was established [42]. As mentioned above, levels of miR34 are dramatically increased in DM, with insulin failing to reduce this augmented expression [29,35]. Thus, by increasing miR34, DM may increase autophagic pathways that could contribute to or precipitate I/R injury.

### 2.2. MiR-144

The role of miR-144 in I/R injury is poorly investigated. However, a recent study suggested a possible role of miR-144 in alleviating diabetic oxidative stress. This study showed that a decrease in miR-144 expression was observed in diabetic cardiomyocytes [43] (Table 1). A similar decrease was also noted in I/R injury in mouse myocardium [44].

MiR-144 appears to aggravate high glucose-induced ROS formation, and this effect is reversed by a Nrf2 activator. Unexpectedly, the expression of Nrf2 was found to be lower in diabetic mice; however, anti-miR144 can reverse this diabetes-induced downregulation of Nrf2 [43]. This is consistent with very recent findings showing that suppressing miR-144 mitigates oxygen-glucose I/R-induced injury via increasing Nrf2 signaling [45].

Although miR-144’s expression was shown to decline in diabetic cardiomyocytes, in vitro experiments showed that the impact of miR-144 on Nrf2 expression was only observed in hyperglycemic conditions, thus reconciling the observations in tissues isolated from T1DM mice [43]. This could potentially implicate other factors being involved in the interplay between miR-144 and Nrf2 expression. Possibly, the downregulation of miR-144 in diabetic cardiomyocytes can be explained as a cellular attempt to respond to oxidative stress by increasing ROS scavengers, albeit to no avail. Significantly, the application of anti-miR-144 was able to decrease ROS levels and attenuate apoptosis following an increase in Nrf2 expression [43]. Henceforth, it may be assumed that the suppression of Nrf2 in DM aids in ROS accumulation and apoptosis in I/R injury. Yet, the exact role and trigger of fluctuations in miR-144 levels remain vague. That said, it is important to note that anti-miR-144 successfully inhibited apoptosis and may thus have a therapeutic potential [43].

Our understanding of the role of miR-144 in autophagy as it pertains to I/R is still in its infancy. It appears that miR-144 can induce pro-autophagic pathways that play a role in cardiac remodeling [44]. This notion is especially noticed when a decrease in miR-144 levels was a key contributor to post-MI maladaptive remodeling [46]. Indeed, this pro-autophagic effect of miR-144 has been shown to improve cardiomyocyte survival [47] and promote cardio-protection [46] (Table 1).

### 2.3. MiR-210

MiR-210 is a silencer of protein tyrosine phosphatase-1B (Ptp1b) [48], which is an activator of both caspase-8 and caspase-3 [49]. In this sense, miR-210 may elicit an anti-apoptotic effect in response to I/R-induced apoptosis. This is supported by the fact that miR-210 was upregulated in response to H_2_O_2_, as an oxidative stressor, in H9c2 rat cardiomyocytes [50]. Other studies lend support to the notion that miR-210 diminishes cardiomyocyte apoptosis induced by oxidative stress [51]. A very recent study reported that the expression of miR-210 is dramatically increased in hypoxic H9c2 cells [52]. Yet this study, in an apparently paradoxical result, reports that when miR-210 is suppressed, hypoxia-induced cardiomyocyte injury was favorably mitigated [52] (Table 1). Regulation of miR-210 expression in response to insulin in the context of I/R injury has also been investigated. Insulin induced the upregulation of miR-210 in H_2_O_2_-treated cardiomyoblasts via the PI3K/Akt pathway [50]. Thus, it could be inferred that in DM conditions where low levels of insulin are present, miR-210 is not upregulated, and thus the I/R injury would be more severe.

In marked contrast to the above-mentioned hypothesis, a study showed that in the absence of insulin, miR-210 increased three-fold in diabetic murine hearts [35]. A possible explanation to the increased miR-210 is the accumulation of ROS in diabetic cardiomyocytes. These cells increased their expression of miR-210 as a protective mechanism against ROS-induced apoptosis. This is supported by other studies showing an increase in miR-210 expression in response to H_2_O_2_ [50]. It is worth mentioning, however, that despite the upregulation of miR-210, I/R oxidative stress remains more critical in diabetic patients [53,54]. This suggests that caspase-8 and caspase-3 may still be activated by Ptb1b, in addition to other molecules, all of which are upregulated in cases of DM.

Overexpression of miR-210 is regarded as a reliable marker of hypoxia [55,56]. In I/R injury models, levels of this miRNA are significantly increased [57]. Autophagy is also reported in I/R injury [58,59,60]. However, whether miR-210 contributes to this autophagy remains undetermined. It is important to mention that a role for miR-210 in promoting autophagy has been suggested in endometriotic cells [61] (Table 1). Whether this is also the case in myocardial tissue, remains to be established.

### 2.4. MiR-141

**Figure 2:**
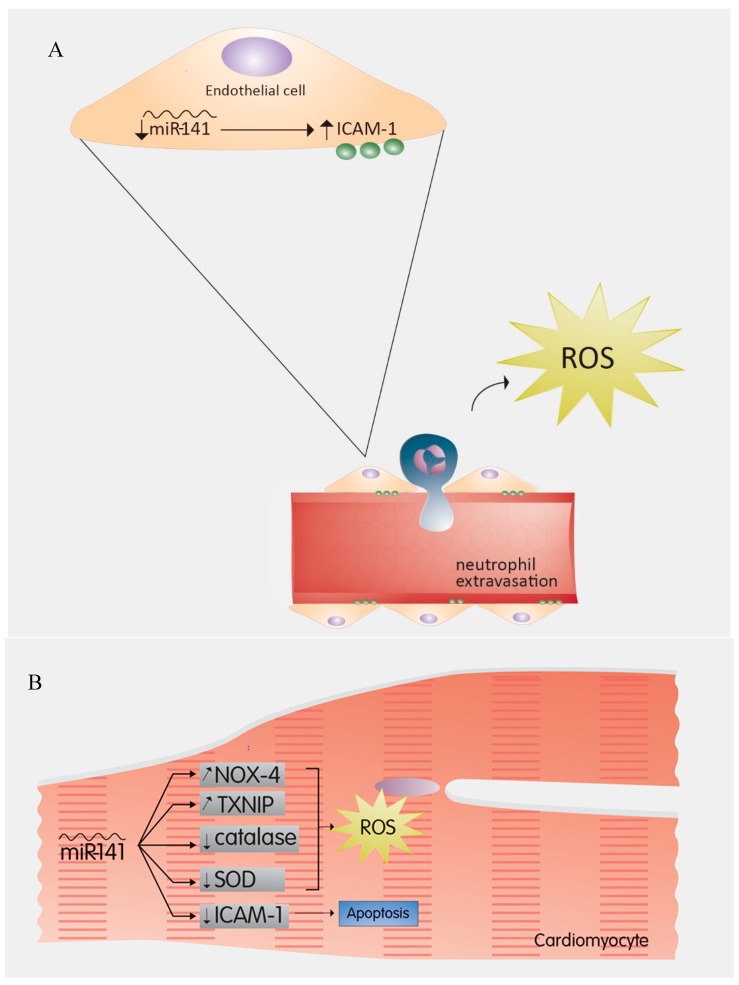
(**A**) miR-141 in endothelial cells: The downregulation of miR-141 and the subsequent upregulation of ICAM-1 results in increased extravasation of neutrophils, which release ROS and amplify necrosis. Hence miR-141 protects endothelial cells in Ischemia-Reperfusion injury. (**B**) Effects of miR-141 on cardiomyocytes: In cardiomyocytes, the upregulation of miR-141 leads to an increase in ROS by increasing the expression of ROS-generating agents such as NOX-4 and TXNIP, as well as decreasing the activity of antioxidants such as catalase and SOD. In addition, miR-141 silences ICAM-1 expression, hence the induction of apoptosis. ICAM-1: Intercellular Adhesion Molecule 1; NOX-4: NADPH Oxidase 4; TXNIP: Thioredoxin-Interacting Protein; SOD: Superoxide Dismutase.

Endothelial cells of cardiac vessels are known to be more prone to apoptosis than cardiomyocytes during I/R injury [62,63,64]. The role of miRNAs in these cells especially under I/R injury conditions is beginning to unravel. In a recent study, miR-141 was shown to play a critical role in the onset of I/R injury by regulating the expression of endothelial intercellular adhesion molecule 1 (ICAM-1) [65]. Treatment of endothelial cells with TNF-α, an ischemia-induced cytokine that promotes the reperfusion injury [66,67,68,69], leads to miR-141 downregulation and ICAM-1 upregulation [65] (Table 1). Administration of exogenous miR-141, however, decreased the expression of endothelial ICAM-1, and subsequently attenuated myocardial injury [65]. This may imply that during I/R injury, the downregulation of miR-141 and the subsequent upregulation of ICAM-1 results in an increased extravasation of neutrophils (Figure 2A). Upon degranulation, neutrophils release ROS and amplify necrosis [70]. This highlights a potential mechanism by which miR-141 imparts protection on ECs in I/R injury.

In the case of DM, miR-141 is upregulated in rat hearts subjugated to glucose fluctuations. Continuous hyperglycemia, like that experienced by hearts of diabetic animals, also evoked a higher expression of miR-141 than normal glucose levels [71]. While this study underscored a beneficial role of miR-141 in the context of non-diabetic I/R, it also showed that the size of the I/R injury was greater in DM hearts compared to controls [71]. The infarct was further amplified when DM hearts were subjected to glucose fluctuations with increasing levels of miR-141 [71]. Such increased levels of miR-141 could promote an increase in the expression of ROS-generating agents, such as NADPH oxidase 4 (NOX4) and thioredoxin-interacting protein (TXNIP), along with a decrease in antioxidant activities of catalase and SOD [71]. The mechanism underlying the amplification of miR-141 and the subsequent effect on ROS-generating enzymes and antioxidants is still unclear but is very likely linked to the cellular O_2_ burst following blood flow restoration to the ischemic tissue. 

The opposing effects that miR-141 has on cardiomyocytes (Figure 2B) when compared to endothelial cells could be due to the notion that miR-141 effect is cell-type dependent with different functions on cardiomyocytes and ECs. Alternatively, it could also mean that despite the fact that miR-141 silences ICAM-1 expression in both cell types, the outcome of such silencing is different and could vary with the presence of comorbidities such as DM. As such, the loss of ICAM-1-mediated cell–cell interaction between cardiomyocytes may underlie the detrimental effects of miR-141 on the myocardium. This is supported by the fact that the loss of ICAM-1-mediated interactions between a cell and its nearby environment promotes cell apoptosis [72]. 

While miR-141 remains an elusive molecule that deserves more in-depth investigation, the two aforementioned papers draw our attention to the necessity of taking the myocardium as a whole into consideration, rather than just conducting experiments on cardiomyocytes only. Although a miRNA may prove beneficial to one type of cells, it may still be harmful to other cells within the same tissue [73], which would hinder its possible therapeutic use. This is especially important in light of recent findings suggesting a role for miR-141 in promoting [74] or inhibiting [75,76,77] autophagy (Table 1).

### 2.5. MiR-155

**Figure 3:**
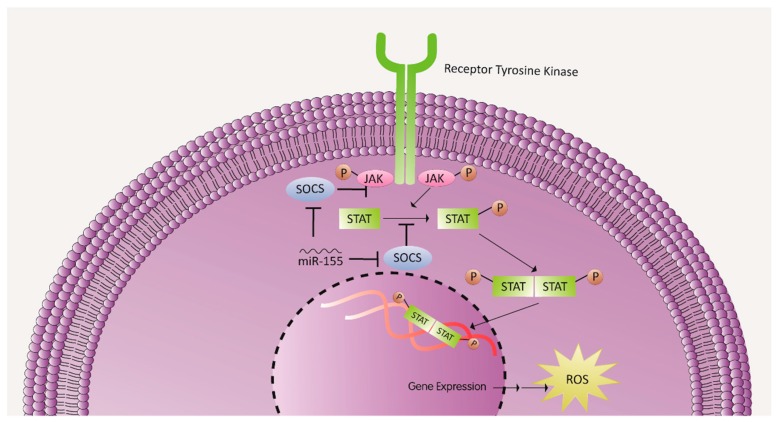
miR-155 role in increased ROS production: miR-155 increases ROS production by silencing SOCS, a negative regulator of the JAK/STAT signaling pathway, a pathway known to play a role in ROS generation. JAK: Janus kinase; STAT: signal transducer and activator of transcription. SOCS: suppressor of cytokine signaling.

MiRNAs have the potential to affect the heart during I/R injury independent of their cell type. For instance, miR-155, which was shown to increase ROS generation under ischemia-induced myocardial hypoxic conditions, is expressed by infiltrating leukocytes [78]. miR-155 upregulation correlated with an increased activation of macrophages, which lead to the overproduction of inflammatory cytokines and chemoattractants, such as interleukin-1β (IL-1β), TNF-α, and monocyte chemoattractant protein 1 (MCP-1) that further recruited more leukocytes to the ischemic heart [78]. In addition, ROS levels in the recruited inflammatory cells following myocardial ischemia were significantly higher than in miR-155-knockout inflammatory cells [78] (Table 1). Mechanistically, this could suggest that miR-155 increases ROS production by silencing suppressor of cytokine signaling 1 (SOCS-1), a negative regulator of the Janus kinase/signal transducer and activator of transcription (JAK/STAT) pathway which is implicated in ROS generation [78] (Figure 3). This hypothesis is supported by two notions. First, there is a notable complementarity between 3’ UTR of the SOCS-1 transcript and miR-155 [79], and second, silencing SOCS-1 or overexpressing miR-155 resulted in similar effects [78]. However, miR-155 may be acting on other upstream or downstream transcripts with higher affinity, and the JAK/STAT pathway may not be the sole signaling pathway affected by miR-155 overexpression. 

In DM, miR-155 is known to be upregulated [80]. Additionally, inhibiting miR-155 is known to reduce cell apoptosis as well as restore cardiac function in a diabetic mouse model [81]. Compromised wound healing that is notable in diabetes is also improved when miR-155 levels are suppressed [82]. Although not studied in the context of diabetic I/R injury, miR-155 inhibition protects against cardiac fibrosis in the diabetic MI mice [83]. Other studies confirmed the pro-fibrotic effect of miR-155 in an MI mouse model [84] or angiotensin II-induced cardiac remodeling [85]. 

In the context of endothelium-dependent vascular relaxation, it was demonstrated that endothelial nitric oxide synthase (eNOS) is a direct target of miR-155 [86]. NO levels impairment and subsequent endothelial dysfunction substantially increased with miR-155 overexpression [86]. Multiple studies linked miR-155 to the progression of atherosclerosis through promoting macrophage-derived foam cells formation, while others showed an M2 polarization protective profile in the miR-155 KO model of viral myocarditis [87,88,89]. All these findings reveal a multifactorial but harmful effect of miR-155 on the cardiovascular system, especially under I/R injury conditions. Although targeting miR-155 might seem a plausible therapeutic strategy, one must consider the multicellular impact of this molecule. Multiple controversial studies have already emerged highlighting the potential protective impact of miR-155 on the cardiovascular system [90]. These include findings suggesting that decreased expression of miR-155 attenuates oxidant-induced injury and upregulates autophagy via modulating ATG5 in endothelial cells [91] (Table 1).

### 2.6. MiR-21 and MiR-146a

**Figure 4:**
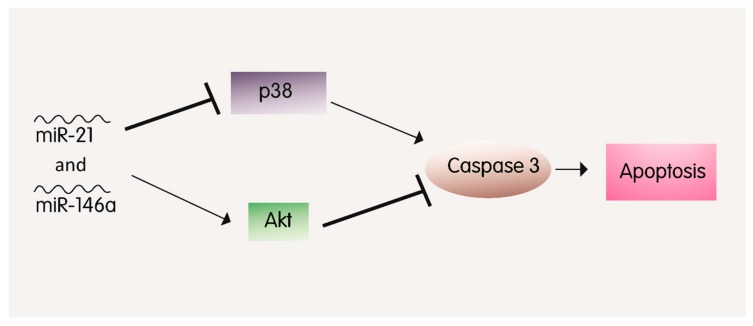
The synergistic role of miR-21 and miR-146a in apoptosis attenuation: miR-21 and miR-146a downregulate p38 and repress Akt inhibition, consequently leading to a decrease in Caspase 3 expression and hence, decreased apoptosis.

Both miR-21 and miR-146a showed independently exhibit a protective role against hypoxia-induced myocardial apoptosis and inflammation in the context of I/R injury [92,93,94,95]. Exogenous miR-146a has been recently shown to be beneficial in promoting the therapeutic potential of stem cells in the I/R-injured heart [96] (Table 1). Consistently, inhibiting miR-146a caused a dramatic deterioration of cerebral I/R injury [97]. Interestingly, the overexpression of both miR-21 and miR-146a in a MI mouse model attenuated apoptosis of cardiomyocytes to a much higher level than the overexpression of each miRNA individually [57]. This synergistic effect might be due to the fact that each of the aforementioned miRNAs inhibits p38-caspase3-induced apoptosis via different mechanisms [98,99] (Figure 4). For instance, miR-21 attenuates cardiomyocyte injury via regulating the programmed cell death 4 (PDCD4) and AKT pathway, whereas miR-146a exerts its protective antiapoptotic effects through modulating interleukin-1 receptor-associated kinase1 (IRAK1), tumor necrosis factor (TNF) receptor-associated factor 6 (TRAF6) and the NF-κB/TNF-α pathways [100,101,102]. This finding opens the window to a potential synergistic effect of multiple miRNAs and their impact on disease prevention or progression [57]. 

In the context of diabetes, both miRNAs are shown to be upregulated under hyperglycemic conditions [103,104,105]. In a myocardial I/R diabetic rat model, miR-21 knockdown exacerbated myocardial apoptosis, thus attenuating the post-conditioning protective effects [106]. Contextually, hydrogen sulfide (H_2_S) protective effects in the context of diabetic myocardial IR were attributed to the upregulation of miR-21 [107]. MiR-146a, on the other hand, has not been studied in the setting of diabetic myocardial I/R, but multiple studies showed a direct modulatory effect of miR146a on the extracellular matrix, apoptosis, oxidative stress, and inflammatory markers expression in the presence or absence of diabetes [108,109,110,111,112]. For instance, it has been reported that in endothelial cells, fibronectin upregulation by glucose is mediated by miR-146a [109]. Similarly, in T2DM rats, increased expression of miR-146a was correlated with decreased inflammation and reduced oxidative stress status [110].

MiR-146 plays an important role in senescence of many cell types. For instance, expression of miR-146a/b or miR-146a is increased in senescent fibroblasts [113] and endothelial cells [114] respectively (Table 1). This increased expression of miR-146a in aortic endothelial cells appears to be associated with a senescent secretory phenotype [114]. Importantly, by virtue of its ability to regulate IRAK in the heart, miR-146a can protect against immune-hyperresponsiveness during I/R injury [115]. Although a role in autophagy has not been reported for miR-21, it appears to protect against cardiac I/R injury by its ability to inhibit excessively augmented autophagic pathways [116]. Consistent with the potential synergy between miR-146 and miR-21, it was recently reported that upregulation of miR-21 is associated with decreased autophagy in myocardial tissues [117].

Taken together, both miR-21 and miR-146a seem therapeutically promising within the context of diabetic I/R. However, one must not overlook their pro-survival effects that could elicit, for example, an adverse fibrotic response or a worsened diabetic retinopathy [108,118,119,120,121]. Indeed, this becomes important since it was very recently shown that downregulating miR-146a-5p may be needed in alleviating I/R injury [122]. Therefore, additional research is still required to better elaborate on the effects of individual and synergetic application of these miRNAs.

### 2.7. MiR-200c

Mir-200 is another well-known family of miRNAs but poorly investigated in cardiovascular diseases. It consists of five members known as miR-200a, -200b, -200c,-141, and -429. Multiple studies recently emerged linking miR-200 to the adverse outcomes of DM, obesity, atherosclerosis, and vascular dysfunction [123,124,125]. Magenta et al. were the first to report that miR-200c expression in endothelial and smooth muscle cells was ROS-mediated [126]. MiR-200c-mediated vascular dysfunction has been linked to its ability to decrease ROS scavengers, increase ROS production, and decrease NO formation [127] (Table 1). Additionally, in the setting of diabetes, miR-200c was directly linked to pro-inflammatory responses and apoptosis of vascular smooth muscle cells (VSMCs) and endothelial cells, respectively [126,128]. This was confirmed by using miR-200c inhibitors that improved endothelium-dependent vasorelaxation of diabetic aortas [129]. 

Although miR-200c is upregulated in the context of ischemia (skeletal muscle) and I/R (brain), its role in myocardial I/R in the presence or absence of diabetes is still largely unknown [130]. However, its levels in diabetics are higher than in control mice [124] (Table 1). In a recent study, miR-200c was linked to enhanced myocardial I/R injury in diabetes, possibly through ROS outburst which was further exacerbated with glucose fluctuation [71]. Of note, not all members of the miR-200 family exert adverse effects. For instance, endothelial miR-200b overexpression prevents diastolic dysfunction and endothelial-to-mesenchymal transition, preserving, therefore, cellular integrity and preventing diabetes-induced adverse myocardial structural and functional changes [131] (Table 1). Importantly, very recently, a report was published showing that levels of miR-200b are lower in patients with T2DM than in control ones [132] (Table 1). Further studies are required in order to elaborate on the different protective and/or adverse impacts that each member of the miR-200 family has in a cell and disease dependent manner in order to optimize any potential therapeutic approach.

### 2.8. Other Promising miRNAs as Therapeutic Targets 

Multiple miRNAs are recently gaining more attention in the context of I/R therapy. In a very recent study, miR-24 that was shown to be decreased in the plasma of diabetic humans, reducing cardiac damage following I/R when overexpressed in the heart of a diabetic rat model [71]. The proposed mechanisms behind those findings were linked to the inhibition of apoptosis, autophagy, and O-GlcNAcylation. MiR-24 has been also linked to apoptosis and fibrosis modulation in cardiac remodeling following a MI [133,134]. Mir-126, another promising target that plays a protective role in I/R vascular homeostasis and angiogenesis, is now known to be downregulated in diabetes [135,136]. Jansen et al. recently demonstrated that patients with diabetes that are at highest risk of coronary artery disease have reduced mir-126 and mir-26a packaging into the endothelial microparticles (EMPs) [137]. In accordance with the potential protective effects, Babu et al. showed that miR-126 overexpression promotes appropriate efferocytosis of apoptotic cardiomyocytes that were otherwise impaired with diabetes [138]. Linking the downregulation of miR-126 EMP packaging with diabetes to the important efferocytotic and angiogenic effects of miR-126, one would expect a worsened remodeling of the heart in the context of diabetic I/R. Further investigation however is still warranted. MiR-17 in another miRNA that has been shown to protect against myocardial [139] and renal [140,141] I/R injury. Interestingly, miR-17 appears to promote hepatic I/R injury via suppressing STAT expression [142]. Last but not least, miR-135a overexpression protected myocardial cells from apoptosis by decreasing TXNIP expression in a diabetic mouse model of I/R [143].

Multiple other miRNAs are gaining more attention in the context of IR injury either due to their potential protective or harmful effects. For instance, miR-133,-146b, 199a-3p, -210, -494, and -499 exert anti-apoptotic effects and protect the myocytes against I/R injury, whereas miR-1, -29,-128, -199a-5p, and -320 promote apoptosis post-myocardial I/R [144,145,146,147,148,149,150,151,152] [153,154,155,156]. Additional experiments are required in order to unveil the underlying mechanisms behind those effects and their role in the context of diabetic IR injury

## 3. Conclusions

In conclusion, the contribution of miRNA to I/R injury in diabetic patients, whether protective or detrimental, is significant. Ostensibly, pro-apoptotic miRNAs are more capable of exercising their effect when compared to the anti-apoptotic ones, most probably due to the diabetic cellular machinery, which is skewed more towards ROS-generating rather than ROS-scavenging. Another probable factor is the presence of extrinsic factors that are upregulating ROS-generating molecules, or downregulating scavengers, diminishing, therefore, the protective effects of miRNAs. Nevertheless, utilization of miRNAs as translational agents or pharmaco-targets in treating I/R injury in diabetic patients could be attractive for future investigations. However, more thorough investigation is needed to elaborate on the effect of specific miRNAs on the system as a whole, rather than on a specific cell type of the studied organ or tissue.

## Figures and Tables

**Table 1:** A list of miRNAs implicated in diabetes that potentially modulate I/R injury via various mechanisms.

miRNA	Function	Level in Diabetes
**miR-34**	silences sirtuin-1 promotes apoptosis induces senescence exacerbates I/R injury	increased
**miR-144**	alleviates diabetic oxidative stress decreases in I/R injury mitigates oxygen-glucose I/R-induced injury promotes autophagy improves cardiomyocyte survival and promotes cardioprotection	decreased
**miR-210**	may elicit an anti-apoptotic effect worsens hypoxia-induced I/R injury increases or decreases apoptosis (depending on model; see text for details) promotes autophagy in endometrial cells	increased
**miR-141**	can promote or inhibit autophagy likely attenuates myocardial injury likely increases ROS in diabetic hearts	increased
**miR-155**	increases ROS worsens cardiac fibrosis in the diabetic MI mouse model promotes autophagy	increased
**miR-21**	protects against hypoxia-induced apoptosis promotes the post-conditioning protective effects in a myocardial I/R diabetic rat model decreases autophagy in myocardial tissue	increased
**miR-146**	inhibits hypoxia-induced apoptosis promotes the therapeutic potential of stem cells in the I/R-injured heart decreases inflammation and reduces oxidative stress induces senescence of endothelial cells inhibits autophagy	increased
**miR-200b**	prevents diabetes-induced adverse myocardial structural and functional changes	decreased
**miR-200c**	increases ROS decreases NO formation contributes to vascular dysfunction is upregulated in ischemia is linked to enhanced myocardial I/R injury in diabetes	increased
